# What are the drivers of female success in food‐deceptive orchids?

**DOI:** 10.1002/ece3.11233

**Published:** 2024-04-18

**Authors:** Ada Wróblewska, Beata Ostrowiecka, Jarosław Kotowicz, Edyta Jermakowicz, Izabela Tałałaj, Piotr Szefer

**Affiliations:** ^1^ Faculty of Biology University of Bialystok Białystok Poland; ^2^ Faculty of Computer Science University of Bialystok Białystok Poland; ^3^ Faculty of Science University of South Bohemia České Budějovice Czech Republic; ^4^ Biology Centre, Institute of Entomology Czech Academy of Sciences České Budějovice Czech Republic

**Keywords:** co‐flowering rewarding plants, *Dactylorhiza*, food‐deception, fruit set, magnet species, orchids, pollinaria removal, remote hypothesis

## Abstract

A large suite of floral signals, and environmental and biotic characteristics influence the behavior of pollinators, affecting the female success of food‐deceptive orchids. In this study, we examined the many factors shaping the reproductive output of three orchid taxa: *Dactylorhiza majalis*, *D. incarnata* var. *incarnata*, and *D. fuchsii*. We applied a statistical model to correlate female success (number of fruit sets) with individual characteristics (plant and inflorescence height, number of flowers, and spur length), number of pollinaria removed, flowering time, and density of floral units of co‐flowering rewarding plants. Our findings suggested that the broad spectrum of variations in *Dactylorhiza's* morphological traits, floral display, and flowering phenology within different environmental contexts has a significant impact on their reproductive success. The number of fruits increased with an increase in the number of pollinaria removed in the studied *Dactylorhiza* taxa. In contrast, a higher number of flowers per inflorescence and higher inflorescences in relation to individual height always decreased fruit set. We observed that low number of co‐flowering rewarding plants in populations could affect the *Dactylorhiza* reproductive output as magnets and competitor plants. The synchronization of flowering, or lack thereof, between *Dactylorhiza* and rewarding plants can limit reproductive success. This demonstrates that the food deception strategy is multidirectional, and reproductive output can vary considerably both spatially and temporally within the context of this strategy.

## Graphical abstract

We observed that a wide range of variations in *Dactylorhiza* morphological traits, floral display, and flowering phenology in an environmental context affects *Dactylorhiza*'s reproductive success. In contrast, a higher number of flowers per inflorescence and higher inflorescences in relation to individual height always decreased female success. The low number of co‐flowering rewarding plants in populations could shape the *Dactylorhiza* fruits set as magnets and competitor plants. The lack of synchronized flowering between *Dactylorhiza* and rewarding plants limits reproduction. This demonstrates that the food deception strategy is multidirectional, and reproductive output can vary considerably spatially and temporally within the context of this strategy.

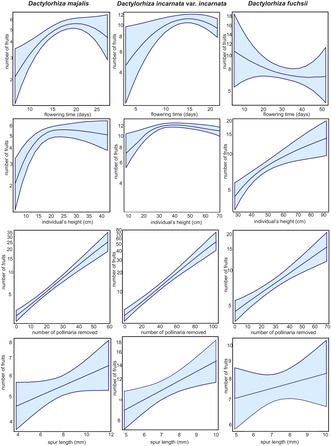

## 1 INTRODUCTION

The food‐deceptive strategy is prevalent in Orchidaceae, where approximately one‐third of orchids do not produce any rewards for pollinators (Cozzolino & Widmer, 2005; Jersáková et al., 2006, 2009; Tremblay et al., 2005). These orchids lure the naïve visitors using the same signals of rewarding plants but dishonestly, i.e. floral displays (such as inflorescence height, number of flowers, their color shape, spur length, etc.) and/or floral scents (Juillet & Scopece, 2010 and references therein; Scopece et al., 2017). Phenological aspects, specifically flowering time and floral longevity, have implications for pollinator behavior in deceptive orchids (Harder & Johnson, 2005; Internicola & Harder, 2012; Sun et al., 2009). Furthermore, interactions between co‐flowering rewarding and deceptive orchids may act as mechanisms for either facilitating or competing for pollination (magnet species and remote habitat hypothesis, respectively; Ferdy et al., 1998; Johnson et al., 2003; Kolanowska, 2021; Peter & Johnson, 2006). In this case, orchids may benefit from the co‐flowering rewarding plants effect or lose their chance for pollination in competition with them. Not all rewarding plants will act as a magnet or competitive species in deceptive orchid populations, therefore, diversity and richness, phenology and flowering duration of rewarding plants can play a key role in orchid reproduction (Capó et al., 2019). Additionally, the spatial distribution, abundance, and density of magnet plants can influence pollinator behavior, usually manifesting as pollinator aggregations which are associated with increased pollination success (Johnson et al., 2003).

While the food‐deception strategy in Orchidaceae has been the subject of extensive examination in numerous studies, it remains still poorly understood phenomenon. The relationships between the floral display traits of orchids, rewarding plants, and reproduction output have been typically described using two‐factor interactions, that is, correlation between fruit set and spur length (Johnson & Steiner, 1997; O'Connel & Johnston, 1998), plant height and flower number (Sabat & Ackerman, 1996; Sletvold et al., 2010), flower brightness (Sletvold et al., 2016) and flower position (Vallius, 2000). However, we still have much to explore in the field of the interactions of multiple factors, which can answer more precisely for level of reproductive success in rewardless orchids (O'Connel & Johnston, 1998; Scopece et al., 2017; Sletvold et al., 2010; Tremblay et al., 2005, 2010).

The *Dactylorhiza* genus is recognized as food‐deceptive orchids, and it has been used as a model for investigating plant–pollinator interactions, natural selection, and, consequent female reproductive success (Mattila & Kuitunen, 2000; Sletvold et al., 2010; Trunschke et al., 2017). The study of Wróblewska et al. (2019) concerning the floral chemical compounds of three *Dactylorhiza* taxa (*D. majalis*, *D. incarnata*, and *D. fuchsii*) revealed the complex multidirectional evolution patterns between them. The differences between these orchids in terms of the concentration of floral chemical compounds, phenotypic traits, time of flowering, and occupation of various habitats provide an opportunity to research female reproductive success in the context of co‐flowering rewarding species. Because the *Dactylorhiza* populations existed in the meadows with a composition of rewarding plants, we could not exclude that these rewarding plants might influence *Dactylorhiza's* reproductive outcomes. Hence, the aim of this study was to identify the effect of several factors such as (1) flowering period, (2) morphology of flowering three *Dactylorhiza* taxa (plant height, inflorescence height, flower number, and spur length), (3) pollinaria removal, (4) diversity of co‐flowering rewarding plants, and (5) the temporal synchronization and spatial density of the co‐flowering rewarding plants on female reproductive success within nine populations of *D. majalis*, *D. incarnata* var. *incarnata*, and *D. fuchsii* over 2 years. We hypothesized that relations between individual and inflorescence height and between flowers and inflorescence height play a crucial role in shaping fruit set. Additionally, we assume the coexistence of both magnet and competing co‐flowering rewarding plants in *Dactylorhiza* populations, and their phenological synchronization with *Dactylorhiza* individuals is crucial in determining reproductive success.

## 2 MATERIALS AND METHODS

### 2.1 Study taxa

*Dactylorhiza majalis*, *D. incarnata* var. *incarnata*, and *D. fuchsii* are presumed to be long‐lived, self‐compatible, perennial orchids that generally reproduce via seeds or occasionally through vegetative means (Ostrowiecka et al., 2019; Vakhrameeva et al., 2008). In Europe, the allotetraploid *D. majalis* complex repeatedly evolved through hybridization between two broadly defined diploid lineages (*D. incarnata* and *D. fuchsii*), inheriting their plastid genomes from *D. fuchsii* (Pillon et al., 2007). They typically produce a single inflorescence and flowers with short spurs (Naczk et al., 2018). The stigma is positioned above the entrance of the spur, while the labellum is notably large and adorned with visual cues (honey guides) that direct pollinators to the spur (Claessens & Kleynen, 2013; Hansen & Olesen, 1999). The pollinaria of *D. majalis*, *D. incarnata*, and *D. fuchsii* were removed in one or two units during a single pollinator visit. In these taxa, the non‐rewarding pollination syndrome is based on food deception (Jersáková et al., 2006, 2009).

Pollination occurs in different taxonomic groups of insects (Hymenoptera, Diptera, and/or Coleoptera, Ostrowiecka et al., 2019; Wróblewska et al., 2019), which can promote cross‐pollination and/or geitonogamy (Hedrén & Nordström, 2009; Ostrowiecka et al., 2019). Moreover, the mechanism of spontaneous caudicle reconfiguration in three *Dactylorhiza* taxa exists, but results in less than 1% autogamous pollination in the studied populations (Tałałaj et al., 2019; Wróblewska et al., 2024). *Dactylorhiza majalis* blooms first, from the beginning of May to the beginning of June. The flowering period of *D. incarnata* var. *incarnata* and *D. fuchsii* is in June and July (Ostrowiecka et al., 2019; A. Wróblewska, personal observation), and fruits occur at the end of July. Variable fruit set is common in the three *Dactylorhiza* taxa, ranging from 7.4% to 77.5% (Claessens & Kleynen, 2011; Kindlmann & Jersáková, 2006; Vallius et al., 2004). These *Dactylorhiza* taxa broadly share the same geographical distribution (Northern and Central Europe and Western Asia) and often grow in sympatry (Tutin et al., 1980).

### 2.2 Study sites

Two‐year studies were performed from May to July in three *D. majalis* populations (KA, 2014–2015; SKI, 2015–2016 and SKII, 2016–2017), three *D. incarnata* var. *incarnata* populations (ZB, 2014–2015; RO and MR, 2015–2016), and three *D. fuchsii* populations (CM, 2015–2016; BR, and GR, 2014–2015) located in northeastern Poland (Table S1, Figure S1). *D. majalis* grows in wet meadows with abundant, entomophilous, and rewarding plants (e.g., *Geum rivale*, *Ranunculus acris*, *R. repens*, *Cardamine pratensis*, *Lychnis flos‐cuculi*, *Trifolium pratense*, and *Alchemilla* sp., which cover ca. 20% of the herb layer in all populations). The abundance of *D. majalis* varied, with around 120–200 (to ca. 1000) flowering individuals (Table S1). The three *D. incarnata* var. *incarnata* populations varied in size, ranging from 35 to 200 flowering plants (Table S1). The populations in the Biebrza and Rospuda Valleys occupied sedge communities with a low cower of the herb layer by rewarding plant species (ca. 10%) such as *L. flos‐cuculi*, *G. rivale*, *Galium palustre*, *Potentilla anserina*, *Oxycoccus palustris*, *Pyrola minor*, *Myosotis palustris*, and *Ranunculus flamula*. *Dactylorhiza fuchsii* was found in open hornbeam forests in the Białowieża Primeval Forest, and nearby areas and one population in the Biebrza Valley (84–193 flowering plants). *D. fuchsii* was observed in an open hornbeam forest, with a low number of rewarding plants (ca. 10%), such as *Anthriscus sylvestris*, *Aegopodium podagraria*, *Vicia sepium*, *Ranunculus lingua*, *Stellaria nemorum*, and *Geranium robertianum* (Table S1).

### 2.3 Data collection and analysis

We assessed population size by measuring the number of flowering individuals, shoot and inflorescence height (in cm), the flower count per inflorescence, and spur length (in mm) on each inflorescence. Additionally, we quantified male reproductive success as the total number of pollinaria removed from each investigated inflorescence in each population and year (refer to Table S1). Female reproductive success was quantified as the number of fruits set per individual per year. We monitored flowering time within each population, systematically observing individuals at 3‐ to 5‐day intervals from the onset of flowering (where nearly all flowers were in the bud stage, with only a single open flower on each inflorescence) to the end of flowering (the perianths had begun to dry out).

In the context of the nine populations, we conducted individual‐scale assessments within 1 m^2^ plots (1 × 1 m) to study flowering *Dactylorhiza* individuals (the number of which varied annually based on population size). Each flowering individual was situated at the plot center, and we recorded the number of co‐flowering rewarding plants. Based on the studies by Carvalheiro et al. (2014) and Henneresse et al. (2017), we standardized flower abundance measurements across all investigated populations and species, defining a floral unit as 1 cm^2^ containing at least one open flower or inflorescence.

For statistical analysis, we treated the three *Dactylorhiza* taxa independently. We employed a negative binomial distribution for the errors with the log‐link function as our statistical model because there was no apparent zero inflation; that is, the number of individuals that did not bear fruit at the end of the flowering period was low. To predict the number of fruit sets, we used eight explanatory variables: (1) flowering time, (2) shoot height, (3) number of flowers per inflorescence, (4) inflorescence‐to‐individual height ratio, (5) spur length, (6) number of pollinaria removed, (7) Shannon's diversity index based on the number of co‐flowering rewarding individuals/shoots, and (8) total number of co‐flowering rewarding plants. We also measured the equivalence factor (*E*) using the formula by Lloyd (1980) as modified by Fritz and Nilsson (1995), *E* = ∑*f*_*i*_/½∑*m*_*i*_, where *f*_*i*_ is the number of fruit produced and *m*_*i*_ is the number of pollinaria removed in individual *i*.

We considered only the quadratic relationship between fruit set and flowering time in our models. The presumed quadratic relationships for the height of an individual, the spur length, and the total number of adjacent flowers were evaluated by comparing models with and without quadratic terms to obtain the simplest model. We included the total number of flowers per inflorescence as an offset term to account for variations in the number of flowers produced by an individual. WAIC‐based backward variable selection was performed for each model to improve the predictive power of our model using the *step* function. At each step of the selection process, we retained the offset term in the formula. Each model was visually checked for goodness of fit, outliers, overdispersion, zero inflation, and variance in the inflation factors (VIF). No clear violations of the model assumptions were observed. Our analyses were conducted using the R statistical software (version 4.2.2, R Core Team, 2022) and the MASS library (Venables & Ripley, 2022).

We also examined the influence of co‐flowering rewarding plant species on the number of fruits set for each *Dactylorhiza* individual. We calculated two measures of flowering synchrony between an orchid individual and its co‐flowering neighbors: (1) Pearson's correlation coefficient between the number of flowers of a focal individual and number of its neighbors within 1 m^2^ to account for the temporal component of synchrony. For example, a correlation close to 1/−1 means that the orchid and its neighboring species bloom synchronously/asynchronously. Values near zero indicate that there is no relationship between the phenologies. (2) Mean difference in flower number between a *Dactylorhiza* individual and units to capture the absolute difference in flower number between a *Dactylorhiza* individual and a particular co‐flowering rewarding plant species. For example, species may flower synchronously, but their neighbor abundance is low. This would result in a positive mean divergence in the flower number. This, in turn, should lead to lower reproductive success in *Dactylorhiza* because there are more false flowers than flowers with true rewards. The mean deviation was standardized (mean subtracted and divided by standard deviation) before analysis. We only used cases in which at least 50 observations were made to ensure the reliable parameterization of statistical relationships.

## 3 RESULTS

**FIGURE 1:**
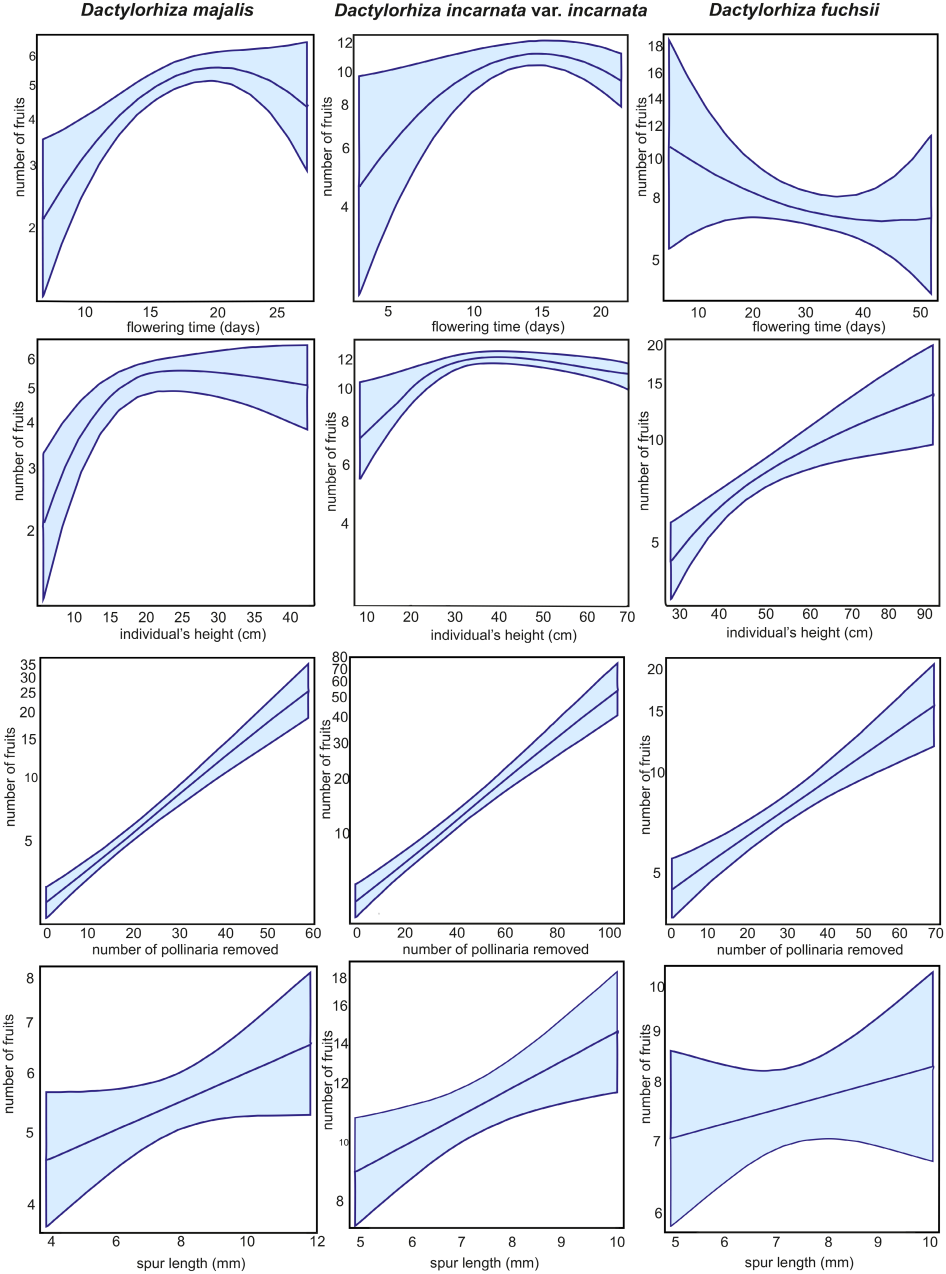
Predicted number of fruits set (partial correlations) for *Dactylorhiza majalis*, *D. incarnata* var. *incarnata*, and *D. fuchsii* as a function of four selected variables: flowering time, the total number of pollinaria taken from a plant, height, and spur length. Full models (see Table 1) were used for the partial correlation plots because we focused on assessing the effects of a predefined set of ecologically important effects.

For all studied *Dactylorhiza* taxa, the positive correlations between the number of fruit sets and the number of pollinaria removed were found to be significant (*p* < .001 in both the full and reduced models; Table 1, Figure 1). Additionally, the ratio of inflorescence height to individual height had negative impact female reproductive success (number of fruit sets) across all taxa (Table 1, Figure 1). Moreover, flower density (number of flowers per inflorescence) and individual's height negatively influenced on the linear predictor of the log‐link function in *D. majalis* and *D. incarnata* var. *incarnata*. Model predictions for each species indicated that the height of the individual reached the highest fruit set at 20 cm for *D. majalis* and at 35 cm for *D. incarnata* var. *incarnata* (Table 1, Figure 1).

**TABLE 1:** Correlation parameters, standard errors obtained from negative binomial models of the number of fruits set per individual of *Dactylorhiza majalis*, *D. incarnata* var. *incarnata*, and *D. fuchsii*.

Taxon	*Dactylorhiza majalis*				*Dactylorhiza incarnata* var. *incarnata*				*Dactylorhiza fuchsii*			
	Full model		Reduced model		Full model		Reduced model		Full model		Reduced model	
Coefficient	Log‐mean	CI (95%)	Log‐mean	CI (95%)	Log‐mean	CI (95%)	Log‐mean	CI (95%)	Log‐mean	CI (95%)	Log‐mean	CI (95%)
Intercept	−3.077***	−4.478 to −1.694	−3.077***	−4.478 to −1.694	−1.398	−2.715 to −0.106	−1.536*	−2.822 to −0.274	−0.285	−1.490 to 0.940	−0.590	−1.301 to 0.114
Flowering time (days) – linear model	0.225**	0.066 to 0.385	0.225***	0.066 to 0.385	0.152*	0.034 to 0.273	0.172**	0.061 to 0.286	−0.027	−0.087 to 0.031		
Flowering time (days) – quadratic model	−0.006*	−0.010 to −0.001	−0.006*	−0.010 to −0.001	−0.004*	−0.008 to −0.001	−0.005**	−0.008 to −0.001	0.000	−0.001 to 0.001		
Individual's height – linear model	−0.018**	−0.030 to −0.005	−0.018*	−0.030 to −0.005	−0.019***	−0.027 to −0.011	−0.018***	−0.026 to −0.010	0.007	−0.001 to 0.015	0.007	0.001 to 0.014
Flowers per inflorescence – linear model	−0.209**	−0.292 to −0.125	−0.209***	−0.292 to −0.125	−0.222***	−0.288 to −0.156	−0.225***	−0.291 to −0.159	−0.233	−0.374 to −0.090	−0.239***	−0.380 to −0.097
Number of pollinaria removed	0.039***	0.032 to 0.047	0.039***	0.032 to 0.047	0.024***	0.020 to 0.029	0.018***	0.020 to 0.029	0.018***	0.013 to 0.025	0.019***	0.013 to 0.025
Inflorescence to individual height ratio – linear model	−2.325***	−3.315 to −1.327	−2.325***	−3.315 to −1.327	−4.273***	−5.749 to −2.938	−4.342***	−5.682 to −2.867	−4.002***	−6.169 to −1.839	−3.784***	−5.857 to −1.639
Spur length – linear model	0.038	−0.004 to 0.081	0.038	−0.004 to 0.081	0.102**	0.027 to 0.177	0.105**	0.030 to 0.180	0.033	−0.043 to 0.110		
Number of units co‐flowering rewarding plant per 1 m^2^	0.001	−0.001 to 0.001	0.001	−0.001 to 0.001	0.000	−0.001 to 0.001		−0.001 to 0.001	−0.003	−0.008 to 0.003		
Shannon's diversity index	0.266***	0.089 to 0.443	0.266**	0.089 to 0.443	0.040	−0.127 to 0.206		−0.127 to 0.206	0.163	−0.083 to 0.412		−0.083 to 0.412
Observation	439		439		191		191		269		269	
*R* ^2^ nagelkerke	.423		.423		.698		.694		.275		.257	

*Note*: The parameters are components of the linear predictor of the log‐link function used in the models. The reduced model represents a model obtained by a simple AIC‐based backward model selection procedure.

*p < .05; **p < .001; *** *p* < .001.

**FIGURE 2:**
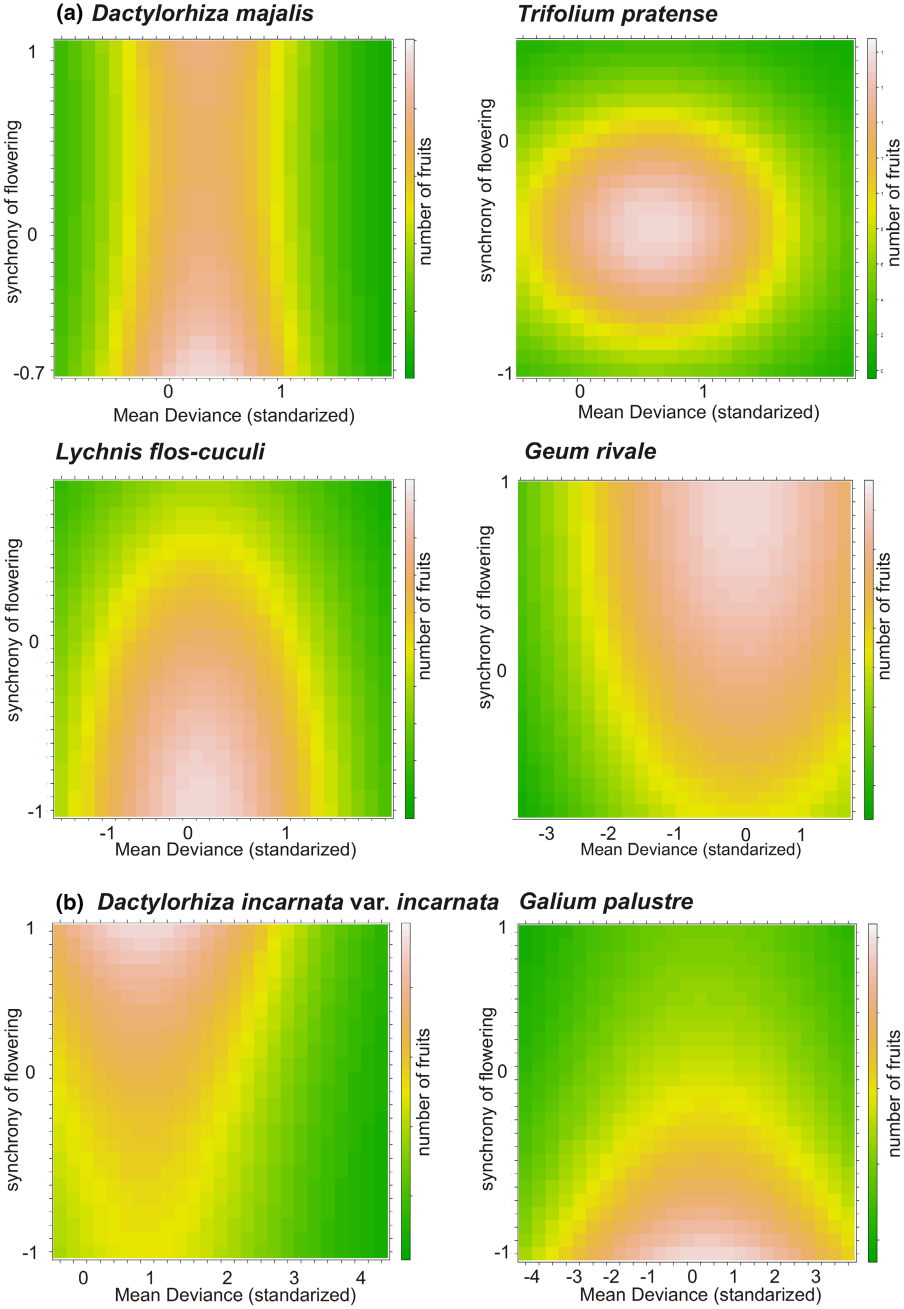
Prediction of the number of fruits set per individual in *Dactylorhiza majalis*, *D. incarnata* var. *incarnata*, and *D. fuchsii* as a function of two parameters describing the effects of synchrony of flowering of adjacent flowers: synchrony of flowering (Pearson's correlation coefficients of the two phenologies) and standardized mean deviance. The mean deviation captures the average difference in the number of flowers/fruits between a focal *Dactylorhiza* individual and a co‐flowering rewarding plant observed simultaneously. The parameters of the fitted negative binomial model (see Table 2) were used to generate predictions for each combination of explanatory variables. The values increase from green to light orange color.

The longest flowering time (average of ca. 30 days) was observed in populations of *D. fuchsia* compared to *D. majalis* and *D. incarnata* var. *incarnata* (average of 20.1 days, respectively, Table 1). Both linear and quadratic parameters for the flowering days were significant only for *D. majalis* and *D. incarnata* var. *incarnata* (Table 1). The levels were highest on days 20–25 of flowering in *D. majalis* and on day 15 in *D. incarnata* var. *incarnata* (Figure 2). Only in *D. majalis*, species diversity increased the fruit set in the full model. In *D. incarnata* var. *incarnata*, the average spur length increased the female success (Table 1). The effectiveness of pollinaria transfer as an equivalence factor (*E*) shaped from low to relatively moderate levels within three *Dactylorhiza* (an average 0.56–0.65; Table S1).

In each *Dactylorhiza* population, we recorded 1–18 co‐flowering rewarding plants (Table S1). Less than 2.4% of these plants were found to play a significant role as either magnet or competing plants in shaping female reproductive success within the populations. In the *D. majalis* populations, we recorded 11–14 co‐flowering rewarding species, although only three of them significantly affected fruit yield (Table 2, Figure 2). Particularly, a higher female reproductive success rate in *D. majalis* was observed when it began to flower synchronously with *Geum rivale* (Figure 2). Conversely, *Trifolium pratense* and *Lychnis flos‐cuculi* began to flower towards the end of *D. majalis* flowering and were noted to interact negatively with this orchid, leading to a significant reduction in fruit yield for *D. majalis*. In *D. incarnata* var. *incarnata* populations, 1–16 co‐flowering rewarding plants were detected, and it was noted that *Geum palustre* acted as a competitor for this orchid (see Table 2, Figure 2, Table S1). The flowering of *D. incarnata* var. *incarnata* and *G. palustre* was noted to be temporally synchronized, with *G. palustre* negatively affecting the female reproductive success of this orchid (Table 2, Figure 2). Notably, no rewarding plants were found to significantly impact the fruit yield of *D. fuchsii*. Additionally, in both *D. majalis* and *D. incarnata* var. *incarnata*, the density of their flowering individuals was found to significantly influence the reproductive success of orchids. The density of *D. majalis* flowering individuals had a negative impact, whereas in *D. incarnata* var. *incarnata*, it positively influenced the fruit set of this orchid.

**TABLE 2:** Results of negative binomial models describing flowering success (number of fruit set per individual) of the three *Dactylorhiza* taxa as a function of synchrony and mean deviation of inflorescences of neighboring plants surveyed at the same time points.

	*Dactylorhiza majalis*		*Lychnis flos‐cuculi*		*Trifolium pratense*		*Geum rivale*		*Dactylorhiza incarnata* var. *incarnata*		*Galium palustre*	
Coefficient	Log‐mean	CI (95%)	Log‐mean	CI (95%)	Log‐mean	CI (95%)	Log‐mean	CI (95%)	Log‐mean	CI (95%)	Log‐mean	CI (95%)
Intercept	−1.155***	−1.309 to −0.999	−1.126***	−1.287 to −0.964	−1.794***	−2.178 to −1.419	−0.859***	−0.987 to −0.731	−1.232***	−1.724 to −0.720	−1.078***	−1.366 to −783
Synchrony	−0.310	−0.623 to 0.003	−0.434**	−0.701 to −0.167	−1.791**	−2.999 to −0.677	0.266**	0.005 to 0.527	0.325**	0.093 to 0.556	−0.488**	−0.828 to −0.163
I Synchrony ^2	−1.296***	−1.851 to −0.749	−0.197	−0.735 to 0.345	−2.733***	−4.306 to −1.245	−0.141	−0.550 to 0.269	0.074	−0.378 to 0.528	0.097	−0.628 to 0.850
Mean Deviance	−0.061	−0.061 to 0.011	0.022	−0.003 to 0.047	0.117	0.037 to 0.199	0.006	−0.007 to 0.019	0.037	−0.010 to 0.082	0.004	−0.005 to 0.013
I Mean Deviance ^2	0.000	−0.002 to 0.001	−0.003**	−0.004 to −0.001	−0.005**	−0.009 to −0.002	−0.001	−0.002 to 0.000	−0.001*	−0.002 to 0.000	0.000	−0.001 to 0.000
Observation	195		253		108		245		92		121	
*R* ^2^ nagelkerke	.285		.130		.268		.048		.221		.136	

*Note*: Synchrony was measured as the Pearson correlation coefficient between the phenologies of a focal *Dactylorhiza* individual and co‐flowering rewarding plant species. The mean deviation was the average difference in the number of flowers between a focal *Dactylorhiza* individual and a co‐flowering rewarding plant.

*p < .05; **p < .01; ***p < .001.

## 4 DISCUSSION

The studies of relationships between the morphological and/or phenological traits and female and male success in food‐deceptive orchids are well‐documented (Mattila & Kuitunen, 2000; Sletvold & Ågren, 2014; Sletvold et al., 2010; Trunschke et al., 2017; Vallius et al., 2007). However, these studies encompass only single‐year data sets and reveal associations such as the impact of floral display on fruit set in *Anacamptis longicornu* (Capó et al., 2019), the density of co‐flowering rewarding plants in *Orchis militaris* (Henneresse et al., 2017), floral display in *Caladenia valida* and *Tolumnia variegata* (Tremblay et al., 2010), inflorescence traits in the environmental context in *Anacamptis laxiflora* (Scopece et al., 2021), as well as flower brightness (Sletvold et al., 2016), flower color (Trunschke et al., 2021), and flowering time (Sabat & Ackerman, 1996). The long‐term study showed only Scopece et al. (2017), who revealed weak directional selection in nine morphological traits in *Orchis mascula* and *O. pauciflora*. At the center of these phenomena are co‐flowering rewarding plants and pollinators belonging to the major selective agents of the floral traits of food‐deceptive orchids (Scopece et al., 2017; Sletvold & Ågren, 2014; Trunschke et al., 2017). Together, these studies have underlined the considerable variability in floral display among food‐deceptive orchids across different populations and years, reflecting a diverse impact on both female and male reproductive success.

In our surveys, we observed a positive correlation between higher numbers of taken pollinaria from inflorescences and increased female reproductive success in both early‐ and late‐flowering individuals of the three *Dactylorhiza* taxa. It was evident that each additional pollinarium removed from the flowers contributed to a higher fruit set. Moreover, the low equivalence factor noted in all our studied populations suggests ineffective transport of pollinaria from one plant to another. This loss of pollinaria by pollinators during flower visits consequently limits the potential fruit set (Harder & Aizen, 2010; Tremblay, 2006). Cozzolino et al. (2005), Tremblay (2006), Capó et al. (2019), and Sun et al. (2018) have argued that the frequency of pollinaria removal, which is higher than fruit set, is the rule rather than the exception in deceptive plants because the flowers act as males but not females. This pattern may arise from a trade‐off between the costs associated with pollinarium production, loss, and fruit set in deceptive plants. This phenomenon has also been emphasized in the context of food‐deceptive orchids by Nilsson (1980, 1983, 1984), Vallius (2000), and Tremblay (2006). Further ecological experiments on *Dactylorhiza* are essential to gain insights into the ecological and morphological factors driving this mechanism.

We have demonstrated that individual height, the ratio of inflorescence to individual height, and the number of flowers per inflorescence were effective predictors for explaining the limitation of pollination success. The observation that shorter *Dactylorhiza* inflorescences and lower flower density on the inflorescence compared to shoot height were associated with a positive effect on female reproductive success in these taxa is particularly intriguing. In the present study, we identified that medium‐to‐short inflorescences in *D. majalis* and *D. incarnata* var. *incarnata*, often yield more outcrossings and higher‐quality offspring. This pattern was observed in other food‐deceptive orchids, such as *Calopogon tuberosus* and *Ionopsis utricularioides*, where fruit set decreases with inflorescence increase (Firmage & Cole, 1988; Montalvo & Ackerman, 1987). Pollinators may quickly learn to shorten their visits to larger inflorescences in multiple‐flowered deceptive orchids when these plants do not offer rewards (Ferdy et al., 1998). Conversely, previous research on deceptive plant species, such as *Dactylorhiza lapponica*, has shown that taller plants with more flowers and longer spurs are favored by natural selection (Sletvold & Ågren, 2014). Pollinators are believed to probe more flowers in plants with larger inflorescences, potentially leading to geitonogamy and negatively impacting reproductive success (Ohashi & Yahara, 2001). These contrasting results demonstrate that the success of the food‐deceptive strategy may depend on a wide range of conditions, with various patterns emerging to optimize reproductive output.

We found that a spur length has a significant impact on the increase of fruit set in *D. incarnata* var. *incarnata*. Conversely, floral chemical components may also have identical pollination service (Alexandersson & Johnson, 2002). We consider the pollinator–flower fit of *D. incarnata* var. *incarnata* on the multi‐faceted interactions, in which both spur length and flower chemical components are affected together in the shaping of orchid sexual reproduction. Fragrance serves a particularly powerful learning cue, playing a key role in pollination of food‐deceptive deceptive *Dactylorhiza* (Wróblewska et al., 2019). It may strengthen associative learning and thus encourage repeat visitation by pollinators (Knudsen & Gershenzon, 2006). Wróblewska et al. (2019) reported that the floral chemical compounds in studied *D. incarnata* populations were dominated by aldehydes and n‐alkanes/alkenes, and the benzoids. The latter ones are ubiquitous in the natural diet of honeybees and might function as a nutraceutical, regulating immune and detoxification processes. They also manipulate the pollinator behavior of *Apis* spp. to learn floral scents more effectively than those rewarded with sucrose alone.

Previous studies have suggested that early flowering in non‐rewarding orchids may be advantageous, as earlier‐flowering orchids are likely to attract more naive pollinators (Castillo et al., 2002; Johnson et al., 2003; Parra‐Tabla & Vargas, 2004; Sabat & Ackerman, 1996; Smithson & Macnair, 1997). Flower duration is also important and can enhance fruit set in deceptive plants (Ruxton & Schaefer, 2009). We did not observe any significant differences in female success in *D. majalis*, *D. incarnata* var. *incarnata*, and *D. fuchsii*, despite different flowering periods. Our study did not confirm the assumptions of Internicola et al. (2006) and Ruxton and Schaefer (2009) that non‐rewarding *Dactylorhiza*, unlike their co‐flowering rewarding plants, should flower earlier and have long‐lived flowers to maximize mating opportunities. We proved that the presence of either only one or multiple rewarding co‐flowering species can significantly affect fruit sets in *D. majalis* and *D. incarnata* var. *incarnata*. Surprisingly, we observed during *Dactylorhiza* flowering, the co‐occurrence of both magnet plants and competitors coexisted within the same orchid population. Our results highlight the complexity of plant‐pollinator interactions and suggest that the influence of rewarding plants on reproductive success may differ among deceptive orchids. Johnson et al. (2003) suggested that nectar plants can be present at a higher density than orchids to achieve the magnet effect. Our results confirmed this statement, and we observed that a higher density of *G. rivale* causes a magnet effect and yielded greater pollination efficiency in *D. majalis*. Our study also shows that the flowering synchrony of *G. rivale* with *D. majalis* had a similar effect. This phenomenon can be interpreted as a strategy to reduce competition with nectar‐producing plants for pollinator visits by *D. majalis* (Johnson et al., 2003; Nilsson, 1980). However, in some species such as *Traunsteinera globosa* (Juillet et al., 2006), *Anacamptis morio* (Johnson et al., 2003), and *Calypso bulbosa* (Alexandersson & Ågren, 1996), low densities of co‐flowering rewarding plants can also act as magnets for pollinators. *Lychnis flos‐cuculi* and *T. pretense*, unlike *G. rivale*, begin flowering at the end of the *D. majalis* flowering period. In contrast, asynchronous and later flowering of *L. flos‐cuculi* and *T. pretense* reduced the female success of *D. majalis*. Interestingly, in this scenario, the density of *L. flos‐cuculi* and *T. pretense* has no effect on the reproductive success of this food‐deceptive orchid.

When *D. incarnata* var. *incarnata* flowered, the density of almost all other rewarding plants was low (*L. flos‐cuculi*, *Geum rivale*, *Galium palustre*, *Potentilla anserina*, *Oxycoccus palustris*, *Pyrola minor*, *Myosotis palustris*, and *Ranunculus flamula*). *Galium palustre* was the only species in this group whose low density and synchronous flowering with *D. incarnata* var. *incarnata* negatively influenced on fruit set of this orchid. Our results suggest that both asynchrony and synchronicity between food‐deceptive orchids and co‐flowering rewarding plants in one habitat can be a critical component of reproductive output in deceptive strategy. In general, contrasting results have been reported between food‐deceptive species, pointing to an environmental context‐dependent reproductive success. The flowering synchrony of deceptive orchids with co‐flowering rewarding plants has not been described, and our research provides the first experimental evidence of these associations.

However, the relationship between density and synchronicity of flowering individuals of *D. majalis* and *D. incarnata* var. *incarnata* at scale 1 m^2^ exhibited diverse patterns. In *D. majalis* medium inflorescence density (0.58–8.9/m^2^ and min‐max 1–19 flower units/m^2^, data not published) has negative female success. Synchronized flowering of *D. incarnata* var. *incarnata* and its low density (0.05–0.8/m^2^ inflorescence and min‐max 1–9 flower units/m^2^, data not published) tended to increase own female success. The number of pollinator visits may decrease faster in *D. majalis* in higher‐density plots of its flowering individuals. However, the low densities of flowering *D. incarnata* var. *incarnata* in 1 m^2^ could increase the number of bumblebees visiting their previous feeding sites when pollinators could not find co‐flowering rewarding species in the population. A similar phenomenon of deception in *Dactylorhiza* pollination has been described by Nilsson (1980) and Vallius et al. (2007).

The generalized food deceptive pollination strategy assumes no specific models and mimics. Indeed, the lack of specialization in pollinators is thought to be the major constraint on mimicry (Schiestl & Johnson, 2013). In the case of three studied *Dactylorhiza*, we observed different groups of pollinators (Ostrowiecka et al., 2019; A. Wróblewska, personal observation). Future studies of an association between deceptive orchids and co‐flowering rewarding plants would be improved by explicit attention to the interpretation of magnet species effect or floral mimicry in three *Dactylorhiza* taxa, separately. Our study did not confirm the floral mimicry but showed that benefits and drawbacks due to co‐flowering rewarding plants in *D. majalis*, *D. incarnata* var. *incarnata*, and *D. fuchsii* population existed. We do not exclude that the flower similarity of deceptive *Dactylorhiza* to the magnet species with which it shares a common habitat is under selection, but it should be tested as the hypothesis (Pellegrino et al., 2008; Scaccabarozzi et al., 2020).

## AUTHOR CONTRIBUTIONS

**Ada Wróblewska:** Conceptualization (lead); data curation (equal); formal analysis (equal); investigation (equal); methodology (equal); project administration (equal); visualization (equal); writing – original draft (lead); writing – review and editing (lead). **Beata Ostrowiecka:** Conceptualization (supporting); data curation (equal); investigation (equal); methodology (equal). **Jarosław Kotowicz:** Investigation (equal); methodology (equal). **Edyta Jermakowicz:** Investigation (equal); methodology (equal). **Izabela Tałałaj:** Data curation (equal); investigation (equal); methodology (equal). **Piotr Szefer:** Formal analysis (equal); methodology (equal); writing – original draft (equal); writing – review and editing (equal).

## CONFLICT OF INTEREST STATEMENT

None declared.

### OPEN RESEARCH BADGES

This article has earned Open Data and Open Materials badges. Data and materials are available at https://zenodo.org/uploads/8094948.

## Supporting information


Figure S1.

Table S1.

## Data Availability

Data and code are provided as private‐for‐peer review via the following link: https://zenodo.org/uploads/8094948.
