# Supplementary material for: What are the drivers of female success in food‐deceptive orchids?
Source: Ecol Evol. 2024 Apr 18;14(4):e11233. doi: 10.1002/ece3.11233 (PMC11026981; doi:10.1002/ece3.11233)
Supplement: Supplementary file 2 — Table S1. [file ECE3-14-e11233-s002.docx]

Table S1. Characteristic of nine *Dactylorhiza majalis, D. incarnata* var. *incarnata,* and *D. fuchsii* populations in relation to locations, morphological traits and female (fruit set) and male success (pollinaria removal); N_CO_ – number of co-flowering rewarding plants in population; N – number of flowering plants; N_F_ – number of individuals of investigated orchid; D_F_ – flowering period; HInd – height of flowering individual; HIf – inflorescence height; Sl – spur length; NF – flower number per inflorescence; F_S_ (%) – fruit set; P (%) – proportion of pollinaria removed; E – equivalence factor (Lloyd 1980; Fritz and Nilsson 1994); x – an average of value; SD – standard deviation. KA – Kapitańszczyzna, SKI – Skupowo I, SKII – Skupowo II, MR – Marachy, ZB – Bagno Ławki, RO – Rospuda, BR – Browsk, CM – Cimoszewizna, GR – Grzędy population.

| Taxon/population/year | | | | GPS location | N_CO_ | N | N_F_ | D_F_ | HInd  (cm ±SD) | HIf  (cm ±SD) | Sl  (mm ±SD) | NF | | | F_S_ (%) | | P (%) | | E |
| --- | --- | --- | --- | --- | --- | --- | --- | --- | --- | --- | --- | --- | --- | --- | --- | --- | --- | --- | --- |
| ***Dactylorhiza majalis*** | | | |  |  |  |  |  |  |  |  |  | | |  | |  | |  |
| KA | 2014 | | | 52°53’00’’N 23°40’29’’E | 11 | ~ 1000 | 126 | 32 | 20.0 ±5.1 | 6.4 ±1.9 | 10.3 ±1.2 | 17.9 ±11.3 | | | 32.3 | | 19.5 | | 0.39 |
|  | 2015 | | |  | 11 | ~ 1000 | 105 | 17 | 26.7 ±6.3 | 7.4 ±2.7 | 8.6 ±1.3 | 19.7 ±10.3 | | | 12.4 | | 49.8 | | 0.64 |
| SKI | 2015 | | | 52°49’50’’N 23°43’10’’E | 11 | ~ 200 | 99 | 19 | 24.1 ±7.3 | 5.2 ±1.1 | 6.7 ±1.4 | 13.1 ±5.6 | | | 35.9 | | 62.2 | | 0.56 |
|  | 2016 | | |  | 11 | ~ 180 | 70 | 15 | 20.4 ±5.9 | 5.1 ±1.6 | 7.2 ±1.1 | 15.1 ±4.7 | | | 50.1 | | 81.6 | | 0.62 |
| SKII | 2016 | | | 52°49’50’’N 23°43’10’’E | 14 | ~ 150 | 70 | 18 | 19.5 ±5.3 | 6.3 ±1.9 | 7.9 ±1.3 | 17.0 ±4.5 | | | 50.6 | | 82.1 | | 0.60 |
|  | 2017 | | |  | 14 | ~ 120 | 80 | 20 | 22.4 ±4.8 | 5.9 ±1.4 | 6.7 ±1.3 | 15.4 ±5.3 | | | 43.3 | | 73.4 | | 0.59 |
| x ± SD | | |  | | | | | 20.1 | 22.4 ±6.4 | 5.8 ±1.4 | 7.8 ±1.2 | 15.9 ±4.3 | | | 37.4 | | 61.4 0.56 | | |
| ***Dactylorhiza incarnata* var. *incarnata*** | | | | | | | | | | |  |  |  | | | |  |  | |
| MR | 2015 | | | 53°47’25’’N 22°57’22’’E | 14 | 100 | 55 | 13 | 24.3 ±4.5 | 8.1 ±8.7 | 8.3 ±0.8 | 26.5 ±8.4 | | | 30.2 | | 39.3 | | 0.76 |
|  | 2016 | | |  | 5 | 68 | 42 | 15 | 18.5 ±6.3 | 4.9 ±1.7 | 6.7 ±1.1 | 20.0 ±8.9 | | | 43.7 | | 88.5 | | 0.47 |
| ZB | 2014 | | | 53°17’59’’N 22°35’44’’E | 6 | ~ 100 | 75 | 24 | 59.3 ±8.3 | 12.7 ±3.2 | 10.3 ±0.4 | 44.8 ±14.7 | | | 16.8 | | 40.2 | | 0.41 |
|  | 2015 | | |  | 1 | ~ 80 | 29 | 28 | 54.3 ±9.4 | 11.3 ±2.9 | 7.5 ±1.5 | 43.7 ±13.3 | | | 10.0 | | 15.7 | | 0.58 |
| RO | 2015 | | | 53°54’39’’N 22°56’32’’E | 15 | ~ 200 | 53 | 23 | 39.3 ±7.5 | 6.9 ±1.6 | 7.3 ±0.6 | 27.8 ±9.2 | | 44.9 | | 70.0 | | | 0.61 |
|  | 2016 | | |  | 16 | ~ 156 | 75 | 18 | 39.9 ±5.7 | 6.6 ±1.2 | 7.1 ±0.8 | 33.4 ±9.6 | | 49.8 | | 91.8 | | | 0.78 |
| x ± SD | | |  | | | | | 20.1 | 39.4 ±6.5 | 8.4 ±1.8 | 7.8 ±1.0 | 35.2 ±8.3 | | 35.7 | | 57.5 | | | 0.60 |
| ***Dactylorhiza fuchsii*** | | | |  |  |  |  |  |  |  |  |  | | |  | |  | |  |
| BR | 2014 | | | 52°50’59’’N  23°53’40’’E | 6 | 102 | 65 | 25 | 40.6 ±7.5 | 7.1 ±2.0 | 6.3 ±0.5 | 21.6 ±6.9 | | | 41.0 | | 62.4 | | 0.69 |
|  | 2015 | | |  | 9 | 133 | 88 | 28 | 43.7 ±10.2 | 8.2 ±3.0 | 7.2 ±0.9 | 22.4 ±8.9 | | | 27.2 | | 51.1 | | 0.53 |
| CM | 2015 | | | 52°41’03’’N 23°39’07’’E | 14 | 122 | 73 | 27 | 45.1 ±7.9 | 7.6 ±2.0 | 7.1 ±1.1 | 25.5 ±9.1 | | | 62.4 | | 74.0 | | 0.77 |
|  | 2016 | | |  | 18 | 84 | 66 | 25 | 46.1 ±9.2 | 7.8 ±2.2 | 7.3 ±1.2 | 24.4 ±7.4 | | | 50.3 | | 83.7 | | 0.60 |
| GR | 2014 | | | 53°36’28’’N  22°50’26’’E | 6 | 140 | 106 | 36 | 58.4 ±11.1 | 8.5 ±2.5 | 7.3 ±1.1 | 22.5 ±7.7 | | | 45.9 | | 53.7 | | 0.83 |
|  | 2015 | | |  | 8 | 193 | 162 | 38 | 53.1 ±7.8 | 8.3 ±1.7 | 7.6 ±1.0 | 22.3 ±7.2 | | | 37.3 | | 75.3 | | 0.49 |
| x ± SD | |  | |  |  |  |  | 29.8 | 48.3 ±11.3 | 7.8 ±1.0 | 7.1 ±0.8 | 23.1 ±9.1 | | | 44.0 | | 66.7 | | 0.65 |
